# Pan-Genomic Analysis of African Swine Fever Virus

**DOI:** 10.1007/s12250-019-00173-6

**Published:** 2019-12-11

**Authors:** Ziming Wang, Lijia Jia, Jing Li, Haizhou Liu, Di Liu

**Affiliations:** 1 CAS Key Laboratory of Special Pathogens and Biosafety, Wuhan Institute of Virology, Chinese Academy of Sciences, Wuhan, 430071 China; 2 Computational Virology Group, Center for Bacteria and Virus Resources and Bioinformation, Wuhan Institute of Virology, Chinese Academy of Sciences, Wuhan, 430071 China; 3 University of Chinese Academy of Sciences, Beijing, 100049 China; 4 CAS Key Laboratory of Pathogenic Microbiology and Immunology, Institute of Microbiology, Chinese Academy of Sciences, Beijing, 100101 China; 5 African Swine Fever Regional Laboratory of China, Wuhan, 430071 China; 6 Center for Biosafety Mega-Science, Chinese Academy of Sciences, Wuhan, 430071 China

Dear Editor,

African swine fever (ASF) is a severe haemorrhagic fever in domestic pigs and wild boar with extremely high mortality rate. It is cataloged as a notifiable disease by the World Organization for Animal Health (OIE). The etiological agent that causes the highly lethal disease is the African swine fever virus (ASFV) (Sanchez-Vizcaino *et al.* 2015). ASFV is the only known member of the genus *Asfivirus* and family *Asfarviridae*. The family *Asfarviridae* belongs to the member of nucleocytoplasmic large DNA viruses (NCLDV) superfamily (Iyer *et al.* 2006; Costard *et al.* 2009). Overall, the ASFV virion presents an icosahedral morphology with a multilayered structure (Wang *et al.* 2019). The genome of ASFV is a large double-stranded DNA (dsDNA) molecule that varies in length from about 170 to 193 kilobase pairs and encodes between 150 and 167 open reading frames (ORFs) depending on the isolate (Dixon *et al.* 2013). In addition, ASFV also infects African wild suids, including warthogs (*Phacochoerus africanus*) and bushpigs (*Potamochoerus larvatus*), which act as asymptomatic carriers. Soft ticks of the *Ornithodoros moubata* complex also serve as a natural reservoir and transmit the disease to suids. In East Africa, ASFV is maintained in an ancient sylvatic cycle involving warthogs and soft ticks (*Ornithodoros* genus) that inhabit their burrows (Jori *et al.* 2013).

ASF was first reported in Kenya in 1921, and now the disease has been endemic for more than twenty sub-Saharan African countries. In August 2018, the first ASF outbreak in China (Shenyang, Liaoning Province) was reported to the OIE. Other Asian countries, such as Mongolia, Cambodia, Vietnam, and North Korea, have reported ASF outbreaks in 2019 (Dixon *et al.* 2019). So far, there is no effective vaccine or antiviral strategy available against ASF. Previous research has shown that variation between the genomes of diverse ASFV isolates results from gain or loss of members of multigene families (MGFs). The families include MGF 100, 110, 300, 360 and 505/530. Additionally, family p22, encoding an early membrane protein, which is adjacent to the left genome terminus exist in some isolates in 1 or 2 copies close to the right genome end (Chapman *et al.* 2008; Dixon *et al.* 2013). Nevertheless, it is still enigmatic which of these genes are requisite and which are secondary, and which are in charge of generality and characteristic during evolution.

Currently, the rapid development of high-throughput next-generation sequencing technologies has facilitated the genomics research. Up to now, more than forty ASFV genome sequences of different origin and virulence are available in public databases. Pan-genomic analysis provides a cogent way to estimate the genomic generality and individuality of ASFV strains, and  to extrapolate the number of additional whole genomes sequences that would be necessary to characterize the overall pan-genome or gene repertoire. The term pan-genome used to describe the entire repertoire of genes or ORFs shared by genomes of interest, including the core genome that contains genes shared by all strains and the dispensable genome made of genes shared by a subset of the strains, and strain-specific genes. The core genome summarizes the generality of all concerned strains of a species and contains the vast majority of genes imperative for life cycle. Dispensable genome and strain-specific genes are viewed as secondary, determining the partially shared and strain-specific characteristics of a species that are not essential to its basic lifestyle (Vernikos *et al.* 2015).

Here, we conducted pan-genome analysis of 42 genomes of ASFV available in GenBank to understand their genomic peculiarity. Firstly, a total of forty-two ASFV genome sequences used in this analysis were retrieved from NCBI GenBank database. The analyzed ASFV genomes were mainly isolated from Africa and Europe, and the avirulent BA71V strain which adapted to proliferate in Vero cells and four isolates from China were also included (Bao *et al.* 2019; Wen *et al.* 2019). These isolates involved a long period of time between 1950 and 2018, and their host mostly cover domestic pigs, wild boars and ticks. The length of entire genomes varies from 170,101 bp to 193,886 bp (GC contents from 38.0% to 38.9%), averaging at 186,817 bp. The majority of isolates possess high virulence, yet the virulence of a few isolates is currently unknown. Viral genomes were annotated using the Genome Annotation Transfer Utility (GATU) (Tcherepanov *et al.* 2006) with the default parameters. The GATU detects all the potential ORFs present in the target and automatically annotates the unknown sequences by using a reference genome.

In previous studies, twenty-four genotypes have been depicted, with the major genotype groups identified based on the C-terminal end of the *B646L* gene coding for the capsid protein p72 (Bastos *et al.* 2003). To determine the genotype of all analyzed strains, the MUSCLE v3.8.31 (Edgar 2004) was employed to align the partial nucleotide sequences of *p72* gene from all strains. Phylogenies were inferred using the maximum-likelihood algorithm in RAxML v8.2.12 (Stamatakis 2014) with 1000 bootstrap replicates. The final phylogenetic tree was visualized using FigTree v1.4.4 (http://tree.bio.ed.ac.uk/software/figtree/). The phylogenetic analysis based on partial *p72* gene sequences revealed that all the 42 ASFV strains were grouped into nine genotypes (Fig. 1A). Notably, the most widely spread genotype was genotype II. This genotype accounted for more than 40% of all ASFV strains, including four isolates circulating in China. The GenBank accession number, strain name, country of isolation, year of isolation, and other features are listed in Supplementary Table S1.

**Fig. 1:**
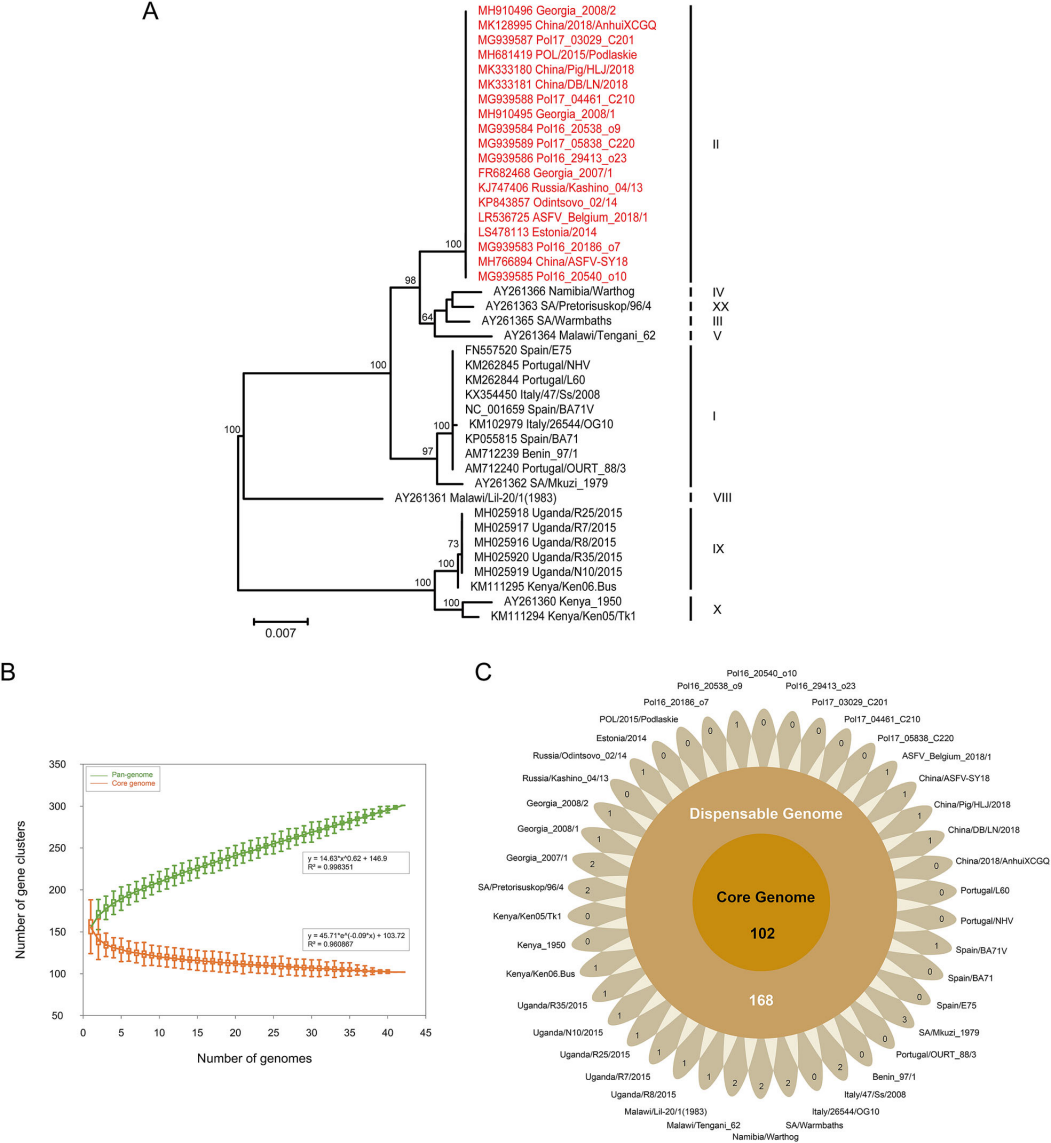
**A** Phylogenetic analysis based on the partial C-terminal *p72* gene. The maximum likelihood phylogenetic tree was inferred with RAxML v8.2.12 using GTRCAT model and 1000 bootstrap replicates. Strains displayed in red emphasize the current wide-spread genotype. The scale bar indicates 0.007 substitutions per site. **B**–**C** Pan-genomic analysis of ASFV. **B** Pan-genome and core genome profiles. The exponent (0.62) of pan-genome curve is greater than zero, indicating an open pan-genome. The curve is the least squares fit of the power law to the average values. **C** Flower plot painting the core genome, dispensable genome, and strain-specific genes of the 42 ASFV strains. The diagram depicts the core gene number (in the center), the dispensable gene number (in the annulus), and the strain-specific gene number (in the petals) for the 42 ASFV strains.

The pan- and core genome analyses of the 42 ASFV genomes were performed using PGAP v1.2.1 (Zhao *et al.* 2012) with Gene Family (GF) method. All protein sequences were aligned using blastall with default parameters (e-value: 1e^−10^; identity: 0.5; coverage: 0.5; score: 40), and ortholog clusters were organized using MCL program. Heaps’ Law model was employed to fit the pan-genome size of strains, and exponential model was applied to fit the core genome size. At last, the characteristic curves of the ASFV pan-genome, the core genome, and the new genes were portrayed using PanGP (Zhao *et al.* 2014) with DG sampling algorithms. Overall, 301 ortholog clusters were identified, which constituted the ASFV pan-genome. The mathematical function was delineated on the graph which shows an exponential value over 0.5, indicating the pan-genome of ASFV is in an open state (Fig. 1B). This trend reflects that ASFV has flexible genome contents, and the size of pan-genome may expand with each added genome which contributes to new genes. As the number of analyzed genomes increases, the core genome curve presents a converging trend. Eventually the number of core genome tends to become a stable value, encompassing 102 ortholog clusters in the core genome (Fig. 1C). The core genome mainly encodes structural proteins, enzymes required for replication and transcription, and factors involved in evading host defense systems and coordinating host cell function. The currently known functions of encoding genes that belong to the core genome are listed in Supplementary Table S2. Furthermore, the ASFV pan-genome also contains 168 dispensable genes and 31 strain-specific genes. These unnecessary genes are deemed to have endowed ASFV with a series of selective advantages in the corresponding environmental niche and the ability to colonize new hosts, resulting in the individuality and diversity of ASFV genome.

To sum up, the investigation revealed the pan-genome of ASFV presented an open state and the core genome was conserved in all of the analyzed strains. An open pan-genome is typical of microorganisms that colonize multiple environments and have multifarious ways of exchanging genetic material (Medini *et al.* 2005). Recent studies have also shown that the homologous recombination contributed much to the genetic diversity of ASFVs (Zhu *et al.* 2019). Illustration of the core genome confers a fundamental understanding of the conservation to ASFV genome during evolution. Our analysis provides a brand-new view on the genomic diversity of ASFV, accelerating our comprehensive understanding of this species.

## Electronic supplementary material

Below is the link to the electronic supplementary material.

Supplementary material 1 (PDF 289 kb)

## References

[CR1] Bao JY, Wang QH, Lin P, Liu CJ, Li L, Wu XD, Chi TY, Xu TG, Ge SQ, Liu YT, Li JM, Wang SJ, Qu HL, Jin T, Wang ZL (2019). Genome comparison of African swine fever virus China/2018/AnhuiXCGQ strain and related European p72 Genotype II strains. Transbound Emerg Dis.

[CR2] Bastos ADS, Penrith ML, Cruciere C, Edrich JL, Hutchings G, Roger F, Couacy-Hymann E, Thomson GR (2003). Genotyping field strains of African swine fever virus by partial p72 gene characterisation. Arch Virol.

[CR3] Chapman DAG, Tcherepanov V, Upton C, Dixon LK (2008). Comparison of the genome sequences of nonpathogenic and pathogenic African swine fever virus isolates. J Gen Virol.

[CR4] Costard S, Wieland B, de Glanville W, Jori F, Rowlands R, Vosloo W, Roger F, Pfeiffer DU, Dixon LK (2009). African swine fever: how can global spread be prevented?. Philos Trans R Soc B Biol Sci.

[CR5] Dixon LK, Chapman DAG, Netherton CL, Upton C (2013). African swine fever virus replication and genomics. Virus Res.

[CR6] Dixon LK, Sun H, Roberts H (2019). African swine fever. Antiviral Res.

[CR7] Edgar RC (2004). MUSCLE: a multiple sequence alignment method with reduced time and space complexity. BMC Bioinform.

[CR8] Iyer LA, Balaji S, Koonin EV, Aravind L (2006). Evolutionary genomics of nucleo-cytoplasmic large DNA viruses. Virus Res.

[CR9] Jori F, Vial L, Penrith ML, Perez-Sanchez R, Etter E, Albina E, Michaud V, Roger F (2013). Review of the sylvatic cycle of African swine fever in sub-Saharan Africa and the Indian ocean. Virus Res.

[CR10] Medini D, Donati C, Tettelin H, Masignani V, Rappuoli R (2005). The microbial pan-genome. Curr Opin Genet Dev.

[CR11] Sanchez-Vizcaino JM, Mur L, Gomez-Villamandos JC, Carrasco L (2015). An update on the epidemiology and pathology of African swine fever. J Comp Pathol.

[CR12] Stamatakis A (2014). RAxML version 8: a tool for phylogenetic analysis and post-analysis of large phylogenies. Bioinformatics.

[CR13] Tcherepanov V, Ehlers A, Upton C (2006). Genome Annotation Transfer Utility (GATU): rapid annotation of viral genomes using a closely related reference genome. BMC Genom.

[CR14] Vernikos G, Medini D, Riley DR, Tettelin H (2015). Ten years of pan-genome analyses. Curr Opin Microbiol.

[CR19] Wang N, Zhao DM, Wang JL, Zhang YL, Wang M, Gao Y, Li F, Wang JF, Bu ZG, Rao ZH, Wang XX (2019). Architecture of African swine fever virus and implications for viral assembly. Science.

[CR15] Wen XX, He XJ, Zhang X, Zhang XF, Liu LL, Guan YT, Zhang Y, Bu ZG (2019). Genome sequences derived from pig and dried blood pig feed samples provide important insights into the transmission of African swine fever virus in China in 2018. Emerg Microbes Infect.

[CR16] Zhao YB, Wu JY, Yang JH, Sun SX, Xiao JF, Yu J (2012). PGAP: pan-genomes analysis pipeline. Bioinformatics.

[CR17] Zhao YB, Jia XM, Yang JH, Ling YC, Zhang Z, Yu J, Wu JY, Xiao JF (2014). PanGP: a tool for quickly analyzing bacterial pan-genome profile. Bioinformatics.

[CR18] Zhu ZZ, Xiao CT, Fan YS, Cai ZN, Lu CY, Zhang GH, Jiang TJ, Tan YJ, Peng YS (2019). Homologous recombination shapes the genetic diversity of African swine fever viruses. Vet Microbiol.

