# Supplementary material for: Pan-Genomic Analysis of African Swine Fever Virus
Source: Virol Sin. 2019 Dec 11;35(5):662–5. doi: 10.1007/s12250-019-00173-6 (PMC7736430; doi:10.1007/s12250-019-00173-6)
Supplement: Supplementary file 1 — Supplementary material 1 (PDF 289 kb) [file 12250_2019_173_MOESM1_ESM.pdf]

VIROLOGICA SINICA

Electronic Supplementary Material

Pan-Genomic Analysis of African Swine Fever Virus

Ziming Wang[1,2,3], Lijia Jia[1,2,3], Jing Li [3,4], Haizhou Liu[2], Di Liu[1,2,3,5,6]✉

1. CAS Key Laboratory of Special Pathogens and Biosafety, Wuhan Institute of Virology, Chinese Academy of Sciences, Wuhan 430071, China
2. Computational Virology Group, Center for Bacteria and Virus Resources and Bioinformation, Wuhan Institute of Virology, Chinese Academy of Sciences, Wuhan 430071, China
3. University of Chinese Academy of Sciences, Beijing 100049, China
4. CAS Key Laboratory of Pathogenic Microbiology and Immunology, Institute of Microbiology, Chinese Academy of Sciences, Beijing 100101, China
5. African Swine Fever Regional Laboratory of China, Wuhan 430071, China
6. Center for Biosafety Mega-Science, Chinese Academy of Sciences, Wuhan 430071, China

Supporting information to DOI: 10.1007/s12250--019-00173-6

Supplementary Table S1. Details of all analyzed ASFV strains from GenBank.

**Virus designation**	**Genbank accession No**	**Country of origin**	**Year of sampling**	**p72 gt**	**Virulence**	**Host**	**Reference**
Portugal/L60	KM262844	Portugal	1960	I	High	Domestic pig	Portugal *et al*. (2015)
Portugal/NHV	KM262845	Portugal	1968	I	Low	Domestic pig	Portugal *et al*. (2015)
Spain/BA71V	NC_001659	Spain	1971	I	Avirulent	Vero cell	Y áñez *et al*. (1995)
Spain/BA71	KP055815	Spain	1971	I	High	Domestic pig	Rodriguez *et al*. (2015)
Spain/E75	FN557520	Spain	1975	I	High	Domestic pig	de Villiers *et al*. (2010)
SA/Mkuzi_1979	AY261362	South Africa	1979	I	Unknown	Tick	Zsak *et al*. (2005)
Portugal/OURT_88/3	AM712240	Portugal	1988	I	Low	Tick	Chapman *et al*. (2008)
Benin_97/1	AM712239	Benin	1997	I	High	Domestic pig	Chapman *et al*. (2008)
Italy/47/Ss/2008	KX354450	Italy	2008	I	High	Domestic pig	Granberg *et al*. (2016)
Italy/26544/OG10	KM102979	Italy	2010	I	High	Wild boar	Bacciu *et al*. (2016)
Georgia_2007/1	FR682468	Georgia	2007	II	High	Domestic pig	Chapman *et al*. (2011)
Georgia_2008/1	MH910495	Georgia	2008	II	High	Domestic pig	Farlow *et al*. (2018)
Georgia_2008/2	MH910496	Georgia	2008	II	High	Domestic pig	Farlow *et al*. (2018)
Russia/Kashino_04/13	KJ747406	Russia	2013	II	High	Wild boar	Unpublished
Russia/Odintsovo_02/14	KP843857	Russia	2014	II	High	Wild boar	Unpublished
Estonia/2014	LS478113	Estonia	2014	II	Low	Wild boar	Zani *et al*. (2018)
POL/2015/Podlaskie	MH681419	Poland	2015	II	High	Domestic pig	Olesen *et al*. (2018)
Pol16_20186_o7	MG939583	Poland	2016	II	High	Wild boar	Unpublished
Pol16_20538_o9	MG939584	Poland	2016	II	High	Wild boar	Unpublished
Pol16_20540_o10	MG939585	Poland	2016	II	High	Wild boar	Unpublished
Pol16_29413_o23	MG939586	Poland	2017	II	High	Wild boar	Unpublished
Pol17_03029_C201	MG939587	Poland	2017	II	High	Wild boar	Unpublished
Pol17_04461_C210	MG939588	Poland	2017	II	High	Wild boar	Unpublished
Pol17_05838_C220	MG939589	Poland	2017	II	High	Wild boar	Unpublished
ASFV_Belgium_2018/1	LR536725	Belgium	2018	II	High	Wild boar	Unpublished
China/ASFV-SY18	MH766894	China	2018	II	High	Domestic pig	Unpublished
China/2018/AnhuiXCGQ	MK128995	China	2018	II	High	Domestic pig	Bao *et al*. (2018)
China/Pig/HLJ/2018	MK333180	China	2018	II	High	Domestic pig	Wen *et al*. (2019)
China/DB/LN/2018	MK333181	China	2018	II	High	Domestic pig	Wen *et al*. (2019)
SA/Warmbaths	AY261365	South Africa	1987	III	Unknown	Tick	Zsak *et al*. (2005)
Namibia/Warthog	AY261366	Namibia	1980	IV	Unknown	Warthog	Zsak *et al*. (2005)
Malawi/Tengani_62	AY261364	Malawi	1962	V	High	Domestic pig	Pan (1992)
Malawi/Lil-20/1(1983)	AY261361	Malawi	1983	VIII	High	Tick	Haresnape & Wilkinson (1989)
Uganda/R8/2015	MH025916	Uganda	2015	IX	High	Domestic pig	Unpublished
Uganda/R7/2015	MH025917	Uganda	2015	IX	High	Domestic pig	Unpublished
Uganda/R25/2015	MH025918	Uganda	2015	IX	High	Domestic pig	Unpublished
Uganda/N10/2015	MH025919	Uganda	2015	IX	High	Domestic pig	Unpublished
Uganda/R35/2015	MH025920	Uganda	2015	IX	High	Domestic pig	Unpublished
Kenya/Ken06.Bus	KM111295	Kenya	2006	IX	High	Domestic pig	Bishop *et al*. (2015)
Kenya_1950	AY261360	Kenya	1950	X	High	Domestic pig	Zsak *et al*. (2005)
Kenya/Ken05/Tk1	KM111294	Kenya	2005	X	Low	Tick	Bishop *et al*. (2015)
SA/Pretorisuskop/96/4	AY261363	South Africa	1996	XX	High	Tick	Zsak *et al*. (2001)

Supplementary Table S2. The currently known gene function of ASFV core genome.

Gene	Function	Function type
A104R	Histone-like structural protein	
A151R	pA151R, component of redox pathway	
A137R	P11.5	
K78R	P10	
B119L	Sulfhydryl oxidase	
B438L	P49, required for formation of vertices in icosahedral capsid	
B602L	Chaperone, involved in folding of capsid	
B646L	P72 major capsid protein, involved in virus entry	
CP2475L	pp220 polyprotein precursor of p150, p37, p14 and p34.	
CP204L	P32 (P30) phosphoprotein, involved in virus entry	Structural proteins and proteins involved in morphogenesis
CP530R	pp62 (pp60) polyprotein precursor of p35 and p15	
O61R	P12 attachment protein	
D117L	P17, required for progression of precursor membranes to icosahedral intermediates	
S273R	SUMO-1-like protease, involved in polyprotein cleavage	
H108R	J5R, transmembrane domain	
E183L	P54 (j13L), binds to LC8 chain of dynein, required for recruitment of envelope precursors to the factory	
E199L	J18L, transmembrane domain	
E248R	k2R	
E120R	P14.5, DNA-binding, required for movement of virions to plasma membrane	
A240L	Thymidylate kinase	
K196R	Thymidine kinase	
F334L	Ribonucleotide reductase small subunit	
F778R	Ribonucleotide reductase large subunit	
G1211R	DNA polymerase	
P1192R	DNA topoisomerase II	
O174L	DNA polymerase X-like	
NP419L	DNA ligase*	
C962R	DNA primase	
E165R	dUTPase	
NP1450L	RNA polymerase subunit 1	
EP1242L	RNA polymerase subunit 2	
H359L	RNA polymerase subunit 3	
D205R	RNA polymerase subunit 5	
C147L	RNA polymerase subunit 6	
CP80R	RNA polymerase subunit 10	Nucleotide metabolism, transcription, replication and repair
A859L	Helicase superfamily II	
B962L	Helicase superfamily II	
D1133L	Helicase superfamily II	
F1055L	Helicase superfamily II	
Q706L	Helicase superfamily II	
QP509L	Helicase superfamily II	
B175L	VV VLTF2-like late transcription factor	
B385R	VV A2L-like transcription factor	
G1340L	VV A8L-like transcription factor	
C315R	TFIIB like protein	
I243L	Transcription factor SII homolog	
C475L	PolyA polymerase large subunit	
D345L	Lambda-like exonuclease	
EP364R	ERCC4 nuclease domain	
EP424R	FTS J-like methyl transferase domain	
NP868R	Guanylyl transferase	
E301R	Proliferating cell nuclear antigen	
A224L	IAP apoptosis inhibitor	
A238L	IkB-like protein, inhibitor of host gene transcription	
CP204L	P32(P30) phosphoprotein	Host cell interactions
EP153R	C-type lectin-like protein	
QP383R	Nif S-like protein	
B318L	Prenyltransferase	
R298L	Serine protein kinase	Other enzymes
I215L	Ubiquitin conjugating enzyme	
